# Fully endoscopic treatment of loss of anatomical continuity of the duodenum caused by a perforated ulcer: an auto-rendezvous by anterograde biliary route

**DOI:** 10.1055/a-2445-8201

**Published:** 2024-11-08

**Authors:** Maria Terrin, Dima Hammoud, Roberta Maselli, Hadrien Tranchart, Ibrahim Dagher, Alessandro Repici, Gianfranco Donatelli

**Affiliations:** 1 Unité d’Endoscopie Interventionnelle, Hôpital Privé des Peupliers, Ramsay Générale de Santé, Paris, France; 2 437807 Department of Biomedical Sciences, Humanitas University, Pieve Emanuele, Italy; 3 Endoscopy Unit, Humanitas Clinical and Research Center IRCCS, Rozzano, Italy; 4 36895 Department of Minimally Invasive Digestive Surgery, Hopital Antoine-Beclere, Clamart, France

Recanalization after total loss of luminal patency in a hollow organ is generally performed surgically, but in unfit patients it may not be feasible, and some reports suggest that endoscopic reconstruction with extra-anatomic rendezvous is a viable option 1 .

An 80-year-old woman underwent surgical suture and drain placement for a duodenal bulbar ulcer. An endoscopic and fluoroscopic evaluation after 5 days showed extensive bulbar necrosis with subversion of the anatomy: we assumed persistence of perforation, with detachment of the second part of the duodenum (D2) from the bulb and from which the drain was visible. A conservative approach was preferred, but unexpectedly the clinical picture rapidly improved: the drain became unproductive and was withdrawn.

At a second evaluation, the bulb appeared fibrotic, without communication with D2, with some minor grooves and a well-defined bifurcated orifice on the superior wall ( Fig. 1 ). Fluoroscopy showed opacification of the biliary tree, but not of D2 ( Fig. 2 ). We explored the bifurcated orifice with a guidewire: the two openings gave access to the intrahepatic and distal biliary trees, respectively, as if the ulcerative process itself had created a “choledochoduodenal anastomosis.” Selective opacification of the “distal” biliary branch permitted transpapillary opacification of D2; this showed no continuity with the bulb but was located near to it ( Fig. 3 ).

**Fig. 1:**
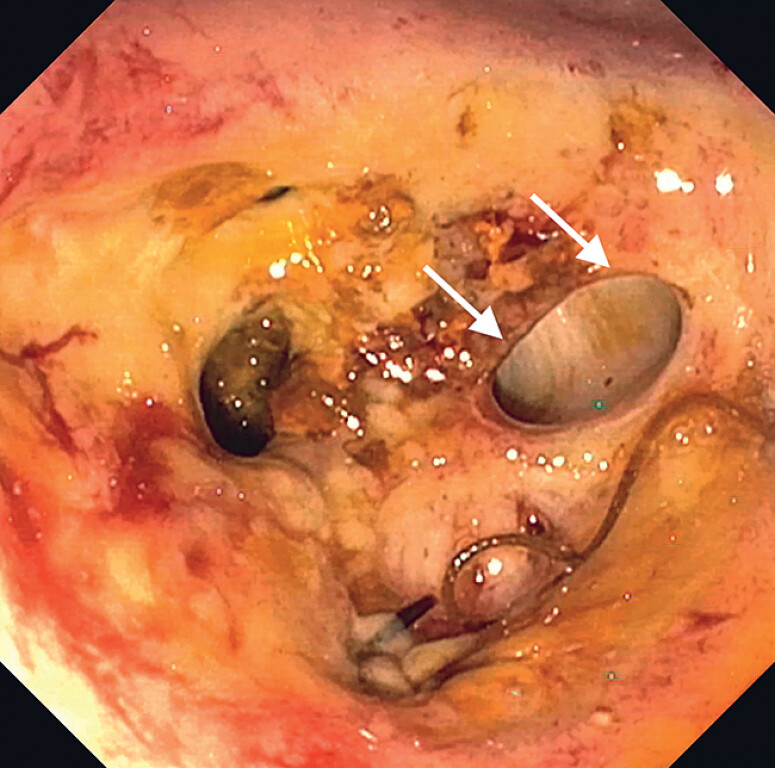
Side-viewing endoscopic visualization of the upper part of the duodenal bulb; several minor grooves and one larger bifurcated fistulous orifice (white arrows) are evident.

**Fig. 2:**
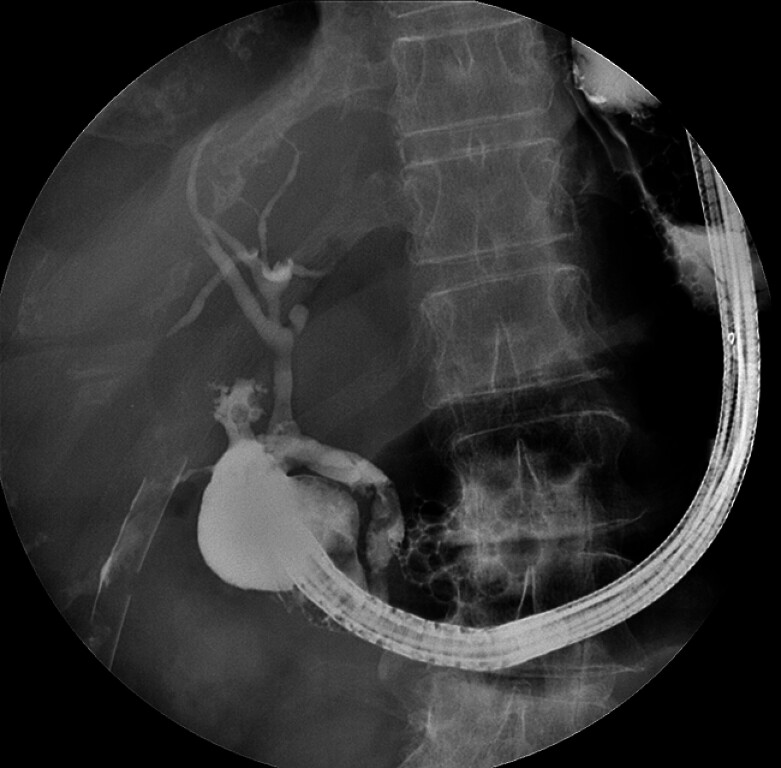
Opacification of the bulbar lumen and of the biliary tree, showing no contrast diffusion to the second duodenal section (D2).

**Fig. 3:**
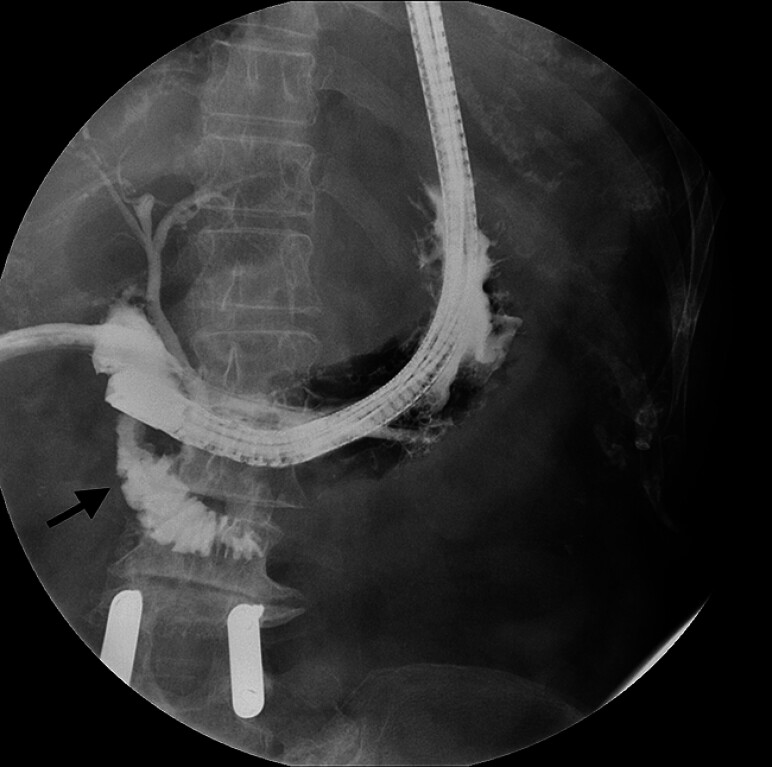
Selective contrast injection into the “distal biliary” orifice in the perforated duodenal bulb results in anterograde transpapillary opacification of D2 (black arrow).

We manipulated a 25-inch double-straight-tipped wire anterogradely through the papilla to D2. The wire was pushed onto the D2 wall adjacent to the bulb, piercing it so that it entered the bulb. The “head” of the wire (now in the bulb) was retrieved through the working channel of the scope, while simultaneously the “tail” was pushed to slide in. The wire now passed through the neofistula in the D2–bulbar walls. The passage was dilated up to 12 mm with a controlled radial expansion (CRE) balloon, and a partially covered metal stent (Hanarostent, 20 mm × 11 cm) was inserted restoring luminal patency ( Video 1 , Fig. 4 ).

Fully endoscopic treatment of bulbar ulcer perforation with obstruction to the second section of the duodenum (D2) by self-rendezvous via an anterograde transpapillary route. CRE, controlled radial expansionVideo 1

**Fig. 4:**
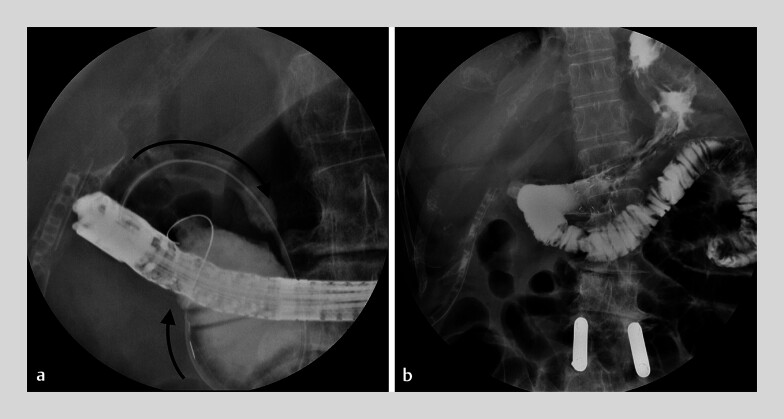
**a** The guidewire forms a loop, passing through the “distal biliary pathway” of the bifurcated orifice, the papilla, and the lumen of D2; then by creating a fistula, the tip re-enters the lumen of the bulb, making a rendezvous with itself (the black arrows indicate the direction of passage of the guidewire). The guidewire tip is retrieved using the working channel of the scope. **b** The duodenal stent in place: the proximal end is in the gastric lumen, the stent passes through the newly created fistula in the bulbar–D2 wall, and its distal end is in the D2 lumen. Contrast injected through the stent flows freely downstream.

Feeding was resumed without discomfort. Subsequent computed tomography confirmed the functionality of the stent, with no fluid or air leakage. We planned an endoscopic re-evaluation in 6 weeks, but unfortunately, the patient died earlier from unrelated disease.

Endoscopy_UCTN_Code_TTT_1AO_2AI

## Notes

Endoscopy E-Videos https://eref.thieme.de/e-videos
*E-Videos* is an open access online section of the journal *Endoscopy* , reporting on interesting cases and new techniques in gastroenterological endoscopy. All papers include a high-quality video and are published with a Creative Commons CC-BY license. Endoscopy E-Videos qualify for HINARI discounts and waivers and eligibility is automatically checked during the submission process. We grant 100% waivers to articles whose corresponding authors are based in Group A countries and 50% waivers to those who are based in Group B countries as classified by Research4Life (see: https://www.research4life.org/access/eligibility/ ). This section has its own submission website at https://mc.manuscriptcentral.com/e-videos .
